# Supplementary material for: In-Silico Evaluation of Anthropomorphic Measurement Variations on Electrical Cardiometry in Neonates
Source: Children (Basel). 2021 Oct 18;8(10):936. doi: 10.3390/children8100936 (PMC8534923; doi:10.3390/children8100936)
Supplement: Supplementary file 1 [file children-08-00936-s001.zip › children-1408532-supplementary.pdf]

children

MDPI

Supplementary Materials

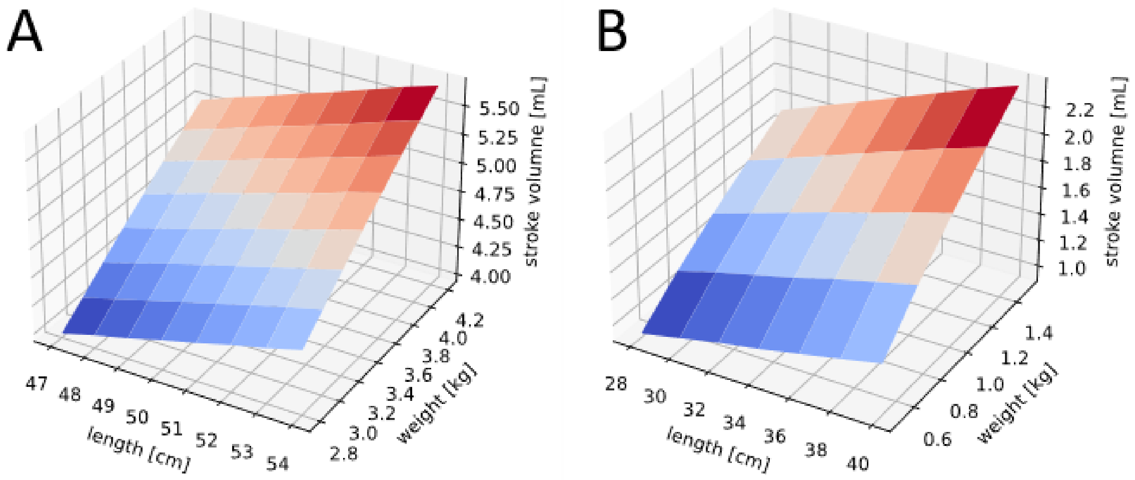

Figure S1. (online supplemental) 3D- Visualisation of raw data (**A**) Term (**B**) preterm stroke volume, length and weight.

Table S1. (online supplemental): Relative stroke volume estimates for term infants.

Length[cm]/ Weight[kg]	3.0	3.2	3.4	3.6	3.8	4.0	4.2
48	85	88	92	95	98	101	104
49	87	90	93	97	100	103	105
50	88	92	95	98	101	104	107
51	90	93	97	100	103	106	109
52	92	95	98	102	105	108	111
53	93	97	100	104	107	110	113
54	95	98	102	105	109	112	115

Stroke volumes represented as a proportion of the result for a term infant of median length and weight (3.6 kg and 51 cm = 100%).

Table S2. (online supplemental): Relative Stroke volume estimates for preterm infants.

Length[cm]/ Weight[kg]	0.5	0.75	1.0	1.25	1.5
28	56	71	83	95	106
30	60	75	89	101	113
32	63	80	94	107	120
34	66	84	100	113	126
36	70	89	105	120	133
38	74	93	110	126	140
40	77	98	115	131	146

Stroke volumes represented as a proportion of the result for a preterm infant of median length and weight (1.0 kg and 34 cm = 100%).
